# A General Outline of the Animal Kingdom, and Manual of Comparative Anatomy

**Published:** 1841-07

**Authors:** 


ART. II.—*A General Outline of the Animal Kingdom, and Manual of Comparative Anatomy.* By THOMAS RYMER JONES, F.L.S., Professor of Comparative Anatomy in King's College, London; Fullerian Professor of Physiology to the Royal Institution, &c. &c. Illustrated by 336 Engravings on Wood.—*London,* 1841. 8vo, pp. 732.

WE have refrained from noticing this work during its somewhat irregularly-periodical issue, because we deemed it better that the whole should be in our hands before we should introduce it to our readers. "Its object," in the words of the author, "is twofold: first, to lay before the naturalist a complete view of the organization and physiological relations of every class of living beings; and, secondly, to offer to the anatomical student a succinct account of the structure and development of the vital organs, through all the modifications they present in the long series of the animal creation." Each class of animals is separately considered; its principal types of conformation being at first sketched, and a detailed account being then given of the structure of the several organs in each of these. The naturalist will here find a rich store of information, in regard to the internal structure of the beings with whose form and classification he concerns himself; on a knowledge of which alone he can safely rely to guide him in his labours. And the physiologist may here trace the development of any system with which he is concerned, through the ascending scale; and may readily acquaint himself with its relations to other systems in each class. The plan is an admirable one, and well calculated to meet the wants of those who desire to gain such a general knowledge of comparative anatomy as may serve them effectually in their more detailed enquiries in either of these allied branches of science.

In regard to the execution of this scheme, we have little but commendation to bestow. Professor Jones has collected his materials from the most authentic sources, judiciously selected those best adapted to his purpose, and arranged and combined them in a manner that exhibits a thorough acquaintance with the principles as well as with the details of his subject. On a few points, however, we do not think that he is quite master of the results of late enquiries, which might have been advantageously introduced. His account of the structure of the polypifera, for example, is encumbered with a redundance of details on some points, in which great simplification has recently been effected; and the classification adopted by him is by no means in accordance with the views of those who have most diligently studied that very interesting group. The embryological details, also, of the development of birds and mammalia, are not modified, as they should have been, in accordance with late enquiries, but follow the imperfect history of some years' date. These are, however, almost the only faults that we have to find with this admirable work; which we can recommend to our readers as adapted, more than any other with which we are acquainted, to furnish them with a competent knowledge of the principal types of conformation encountered in the study of the animal kingdom. The wood engravings are beyond all praise. With the exception of Wilson's Manual of Anatomy, we have never met with a work so richly illustrated; and our only regret is that its price must keep it out of the reach of many who would otherwise have been glad to possess it.

